# Aggravation of Human Diseases and Climate Change Nexus

**DOI:** 10.3390/ijerph16152799

**Published:** 2019-08-06

**Authors:** Mohd Danish Khan, Hong Ha Thi Vu, Quang Tuan Lai, Ji Whan Ahn

**Affiliations:** 1 Resources Recycling Department, University of Science and Technology, (UST), 217, Gajeong-ro, Yuseong-gu, Daejeon-34113, Korea; 2 Center for Carbon Mineralization, Mineral Resources Research Division, Korea Institute of Geosciences and Mineral Resources (KIGAM), 124 Gwahak-ro, Yuseong-gu, Daejeon-34132, Korea

**Keywords:** climate change, infectious diseases, pathogens, vectors, human adaptation

## Abstract

For decades, researchers have debated whether climate change has an adverse impact on diseases, especially infectious diseases. They have identified a strong relationship between climate variables and vector’s growth, mortality rate, reproduction, and spatiotemporal distribution. Epidemiological data further indicates the emergence and re-emergence of infectious diseases post every single extreme weather event. Based on studies conducted mostly between 1990-2018, three aspects that resemble the impact of climate change impact on diseases are: (a) emergence and re-emergence of vector-borne diseases, (b) impact of extreme weather events, and (c) social upliftment with education and adaptation. This review mainly examines and discusses the impact of climate change based on scientific evidences in published literature. Humans are highly vulnerable to diseases and other post-catastrophic effects of extreme events, as evidenced in literature. It is high time that human beings understand the adverse impacts of climate change and take proper and sustainable control measures. There is also the important requirement for allocation of effective technologies, maintenance of healthy lifestyles, and public education.

## 1. Introduction

Climate change is a significant statistical shift in regional or global climate variables over a considerable period of time (decades or even more). The most sensitive climatic variables include precipitation pattern, humidity, average and peak temperatures, and wind [1]. Although the global mean temperature and other climatic variables were relatively stable for millennia, recent decades have witnessed considerable changes [2]. The European Environment Agency reported a rise of 0.74 °C in global mean temperature in the 20th century, a continuous 1.8 mm per year expansion of sea level since 1961, and a 2.7% reduction in Arctic sea ice per decade [3]. Climate experts have also confirmed the dramatic increase in the frequency and severity of extreme weather events over the last few decades. Moreover, a remarkable rise of 1.5 °C to 5.8 °C in global mean temperature was predicted by the Intergovernmental Panel on Climate Change (IPCC) in the 21st century, accompanied by more severe weather events dominated by floods and droughts [4,5]. Climatologists have also provided very clear evidence that even a short-term climatic variation can pose serious threat to human health in many different ways.

Climatic variations can significantly influence human health through the emergence of various infectious diseases [6,7,8,9]. Pathogens, vectors, and favorable transmission conditions are the three essential components involved in most infectious diseases [7]. Optimum climatic conditions are a prerequisite for reproduction, survival, mortality, and spatiotemporal distribution of vectors and disease-causing pathogens. Therefore, a sudden or long-term climate change can have a positive impact over infectious diseases [7,10]. Numerous studies have divulged the continual impact of rising rates of warming on the geographic expansion of pathogen-based diseases [11,12,13,14,15,16,17,18,19]. Studies also have found that increased global temperature boosted skin-related diseases [20]. More cases and hospital admissions have been reported regarding cutaneous injuries from marine and aquatic organisms that have expanded their habitats due to favorable warm water environments, such as cercarial dermatitis, jellyfish envenomation and melioidosis [21,22]. Furthermore, extreme weather events can also motivate the emergence and re-emergence of clustered diseases in many other non-traditional regions [6]. Conclusively, climatic conditions modulate the seasonal and geographic distribution of vector-borne diseases, and weather influences the severity and timing of disease outbreaks [10,23].

The inter-relationship amidst contagious pathogenic activities, vectors’ susceptibility, and climatic variations have been a topic of great interest. As an example, any modulating factor that prolongs the ‘stochastic trends of disease transmission’ also permit pathogens to acclimate to the intermediate host and aggrandize disease evolution [24]. Being an inherent module of this rubric, climate change act as a background context and if changed, affects the susceptibility and transmission of infectious diseases. This research put forward a systematic literature review covering numerous scientific evidences, which highlight the effects of climate change and weather events on human-based diseases. Precisely, the present study provides a reconnaissance for the scrutinized and predicted impacts of climate change and weather events over disease-causing pathogens and vectors. The research also underlines the need for future studies by discussing the voids in research progress, productive policies and human adaptation.

## 2. Methods

**Figure 1:**
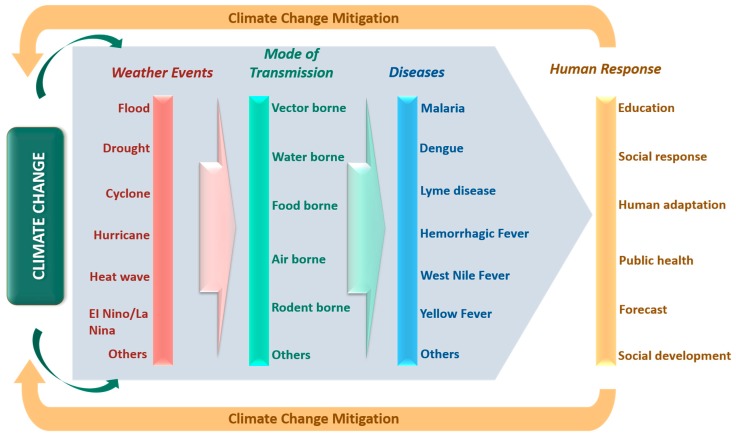
The relationships between climate change, extreme weather events and associated diseases with their mode of transmission. It also further represents possible urgent human responses for mitigation of climate change adapted from [1,2,3,4,5,6,21,22,23,24].

As discussed earlier, the consequences of climate change on a variety of human diseases can be well examined based on two components: Emergence and re-emergence of pathogenic diseases, vectors, and favorable conditions for transmission and infectious disease outbreaks post weather events. Moreover, as the discussion is in context of human beings, human response and adaptation efficacy can be vital in mitigating the adverse impacts of climate change. The interconnections between climate change, extreme weather events, diseases, and human responses, forming the rationale for the present literature search, are illustrated in Figure 1.

Literature searches in the present work were conducted through ScienceDirect, PubMed, PLOS, Google Scholar, Nature.com and MDPI. Furthermore, published reports provided by IPCC, UNICEF and WHO were also reviewed, where required. The primary focus was to collect peer-reviewed literature published between 1990 and 2018 to gather maximum scientific facts and evidences. 

The Medical Subject Heading (MeSH) terms searched in PubMed, ScienceDirect and PLOS were: Viral infections, bacterial infections, pathogens, vectors, parasites, rodents, infectious diseases, diseases, skin diseases. “PECOT” terms included population, exposure, comparison, outcome and time. Climatology terms included temperature, humidity, wind, rain, precipitation, climate, climatic, extreme weather; extreme weather events were also searched. Other keywords such as Africa, Asia, Europe, South America, Australia, Canada, United States, policies, adaptation, population, exposure, human, response, education, public health, social response, outcome and time were also searched. The mentioned terms were coherent with those used in the previous review [25], but additional terms such as “policies,” “adaptation,” “social response,” “ocean warm-up,” “time,” “exposure,” and “population” were added. MeSH terms were preferred rather than individual keywords, as used in previous reviews [25,26]. As MeSH terms were used for the PubMed, ScienceDirect and PLOS searches, keywords contained significant phrases in the MeSH hierarchy. For instance, terms such as malaria, dengue, yellow fever, lyme, encephalitis and zoonoses are all included in “infectious diseases”, a hierarchical MeSH phrase. The term “zoonoses” was not searched separately, rather the phrase “zoonotic diseases” was used because viral and bacterial infections and parasitic diseases all fall within their MeSH hierarchy.

For literature searches in Nature.com, Google Scholar, MDPI and government reports, the following keywords were used: infectious diseases, pathogens, vectors, viral infection, bacterial infection, extreme climatic events, climate change, temperature, precipitation, ocean warm-up, disease transmission, policies, adaptation, human, response, education, public health and social response. The mentioned searched terms were precisely chosen to explore across databases and to fulfill the selection criteria as much as possible.

Although numerous literatures were searched across databases, the only limitation found was the unavailability and security issues in accessing recent government reports with proper facts and figures for an individual country based on ‘impact of climate change on human diseases’. Economic constrains were another factor that hindered any possible access to relevant government official databases. 

The search criteria were based on the following requirements: (a) articles and reports with a considerable correlation between the climatic variable and human diseases. The source of those diseases can be a pathogen or a vector/host whose survival or activities can be significantly influenced by climatic factors. (b) Climate change and required human response. The human response can involve relevant policies, education, adaptation measures and social response. No region-wise restriction and data from across the globe was considered. All searches were conducted from July 2018 onwards till 30 November 2018. 

**Figure 2:**
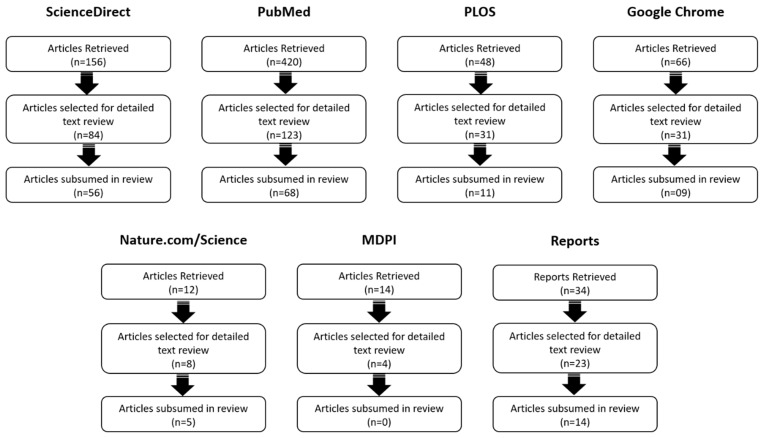
Illustration of the article-screening process across databases: Articles retrieved, articles selected for detailed text review, and articles subsumed in review (where n denotes the number of articles/reports).

All the search terms used, and the number of results retrieved for each search in various databases were recorded. To determine each article’s relevance, their titles and abstracts were carefully evaluated. All the articles that fell within the criteria were extracted for a detailed text review. Those articles that met the selection criteria post detailed text reviews were included in the present work. All duplicate articles in different databases were counted as single articles and were selected from the first database they were found in. Figure 2 depicts the numbers of search outcomes, reviewed articles and articles included in this review across databases. 

Initially, more than 750 peer-reviewed publications were retrieved from databases. After title and abstract evaluation, 300 articles were selected based on inclusion criteria. Finally, post full text review analysis, a collection of 163 articles and reports were schematically included in the present work. Those articles and reports comprehensively focus on numerous consequences of climate change over human diseases and highlight the urgent requirement for a human response.

## 3. Extreme Weather Events

Extreme climate/weather events represent the outcome of climatic variables that breach the threshold limits [27]. On a broader basis, these events can be categorized as global scale extreme events like El Nino, La Nina and Quasi-Biennial Oscillation (QBO), and regional scale events such as floods, droughts, and shift in precipitation and drought. Regional events are very rare (<5% of time) but their intensity and frequency are rising significantly, representing the adverse impact of climate change [27,28,29]. Extreme weather events may not only result in the huge spread of diseases through the increased growth of vectors, pathogens, viruses, and transmission routes, but can also cause a breakdown of public health infrastructure, loss of sanitation and hygiene, shortage of drinking water supply, and increased concentration of people.

Many studies have been conducted on the consequences of global and regional weather events on human diseases, as depicted in Table 1. Majority of these studies are empirical rather than comprehensive. They lack the real mechanism between climate change and various diseases and often contradict each other. As an example, a study by Nicholls revealed that in Peru, Ecuador and Bolivia, the outburst of malaria was due to heavy rainfall caused by an El Nino event around 1983 [30]. However, it was also found that there is no link between hospital admission and climate change based on malaria in East Africa under the El Nino event [31,32]. Another study found a sudden downfall in malaria cases in regions of the Usambara Mountains, Tanzania after the 1998 El Nino event. Similar contradictions were found for the case of Rift Valley Fever (RVF) epidemics. In the dry grassland regions of East Africa, heavy rainfall was found responsible for the epidemics of RVF [33,34]. However, in Kenya, no association was identified between El Nino and RVF and also no correlation was noted between heavy rainfall variability and El Nino [33].

Many researchers have claimed climate change to contribute to cardiopulmonary disorders such as acute coronary syndromes, infarction and other similar mortalities [35,36,37,38,39]. Although climate change may seem to provide some health benefits (in early stages) in such disorders [1], they pose much more health hazards. A warmer climate produces much intense heat waves on a frequent basis, the negative effects on which on human health are well-known [40]. Incidences of cardiovascular and respiratory diseases in children as well as in adults have increased tremendously from the last century [41]. A close relationship had been found between hospital admission, environmental temperature, and humidity for angina pectoris [42]. Another research in the US revealed that humidity with higher temperatures resulted in an increment of asthma patients’ visits to hospitals [43,44]. Numerous model projects based on the climate in Europe and North America provided evidence for 50% increment in intensity and frequency of hottest days in the 21^st^ century [45,46]. In the US, more than 400 deaths are reported annually due to excessive heat-related sickness [47]. Recent studies also confirmed the association between higher temperature and myocardial infarction along with congestive heart failure due to excessive heat [48,49]. A detailed understanding of change in climate patterns and combined climate effects on human health is highly desirable. This not only provides support in predicting health effects from weather-related events but also plays a potential role in effectively controlling many diseases. 

Variations in the magnitude of weather events might also be responsible for a variety of health effects. For example, a study conducted by Chen et al. found that cases of Hemorrhagic Fever with Renal Syndrome (HFRS) increased during the floods in 1954, 1975 and 1991, implicating HFRS with floods directly [50]. However, in the flood incident of 1998, HFRS cases reduced significantly, contradicting previous findings. Finally, further research revealed that the rodent population was ultimately responsible for HFRS, which reduced greatly in regions affected by floods in 1998. A similar kind of study was performed by Pan et al. in which the main reason for HFRS was related to variable water levels in flooded areas [51]. Some studies were also performed on weather events such as hurricanes and cyclones [6,52,53]. The aftereffects of those events on human health were seen as a widespread increase of malaria, dengue, leptospirosis, and cholera. Lastly, heat and drought are mainly responsible for wildfire in many parts of the world. Smoke from wildfires can cover a wide area exposing people to hazardous gases and particulate matters [54,55]. The above examples illustrate the lack of enough data as well as contradictions among studies, highlighting the limited ability to predict diseases and any change related to their course or intensity during different weather events. Hence, there is the utmost requirement to accurately predict all possible changes in climate variables that are expected to be associated with extreme weather events. 

## 4. Relationship Between Climate Change and Transmissible Diseases

Climate change can be directly or indirectly detrimental to human health. To date, a majority of the investigations were confined to direct effects of climate change; for instance, heatwaves, floods, drought, hurricane, cyclone and other extreme weather events [27,61]. It was recently identified that the indirect effects of climate change in terms of infectious diseases are also severe and unlike direct effect could last for a longer period of time over a much wider region [37,38]. Climate change can alter environmental temperature, wind, and precipitation patterns, which can indirectly affect pathogen distribution, vector reproduction rates, and transmission media. These effects then further determine regional shifts in seasonal patterns of individual diseases along with its respective frequency and severity. In this section, the impacts of climate change over infectious diseases have been systematically surveyed primarily over impacts on pathogens, vectors or hosts, and mode of transmission. Empirical findings related to pathogens, vectors and associated diseases are summarized in Table 2.

### 4.1. Impact on Pathogens 

Pathogens refer to a broad variety of disease-causing agents such as bacteria, viruses, fungi and other microorganisms, and parasites. Till date, there are more than 1400 known species of human pathogens. These pathogens are mostly transmitted to humans either zoonotically (60%) or other environmental mediums (~10%). Zoonotically transmitted pathogens require an animal body as their habitat for survival. The remaining pathogens can be directly transmitted human-to-human [62]. The emergence and re-emergence of zoonotic infections are much higher (around 73%), representing the associated risk of human-animal interface [63]. These pathogens can be affected with the changes in climate directly through alterations in reproduction mechanism and survival behavior. Indirectly, it can affect by influencing the environment, particularly their habitat. Therefore, climate change can potentially enhance quantity and can also induce a major shift in the distribution of pathogens [26].

Temperature has a most significant impact on transmission and life cycle of pathogens. In a review study by Mellor and Leake, it was concluded that environmental temperature greatly controls the transmission of Japanese Encephalitis Virus (JEV) [64]. For example, at 28 °C, transmission of JEV by *Culex. tritaeniorhynchus* was 60% and 100% after 9 and 14 days, respectively. However, at 20 °C, no transmission was recorded, even after 20 days of monitoring. Another study also highlighted that threshold temperatures i.e., a maximum of 22–23 °C for developing mosquitoes and a minimum of 25–26 °C for transmission of JEV determines the ecology of JEV [65]. Excessive temperature rise can also favor pathogens in their reproduction mechanism in terms of the extrinsic incubation period (EIP) [11]. For instance, in the case of *Plasmodium falciparum*, it is found that at 20 °C the EIP is around 26 days, which reduces to only 13 days at a temperature of 25 °C [66]. During summer periods, an increase of about 40% infections associated with *Enterobacter cloacae* was also found [67]. A similar dependence of increasing temperature on *Klebsiella pneumoniae* bloodstream infection was also investigated by Anderson et al. [68]. A positive relationship between warm wet season and growth of *Acinetobacter* spp. related infectious diseases were also revealed by Allen at al. [22]. There are also some positive implications of extreme heat and mortality rates for some pathogens [23]. For instance, the growth of parasites such as *Plasmodium* spp. associated with malaria minimizes when temperatures surmount 38–39 °C [69]. Global warming might also induce genetic modifications in pathogens like chikungunya viruses, which might make them more resilient and reproduce at faster rate [70]. Extreme hot weather is reported to have adverse impacts on aquatic environments, often leading to an exponential rise in microorganisms. It was found that the growth rate of *Vibrio* spp. was much higher on hotter days when compared to normal days [71]. A positive correlation has been identified between *Salmonella* spp. growth and water temperatures, whereas in *Escherichia coli*, it was negative [72,73]. El-Fadel et al. developed a Poisson generalized linear model to identify and predict the relationship between water- and food-borne morbidity rates and associated high temperatures [74]. It has been observed that morbidity rates decrease to a threshold temperature of 19.2 °C and then increases with temperature. It has also been predicted that by the year 2050, water and food-borne morbidity rates will rise to 28%, which can further increase to 42% by the end of 2100. 

Climate change can also significantly influence the precipitation pattern and humidity level, which in turn could provide a beneficial environment for some water-borne and air-borne disease pathogens. Considerable rain after dry periods can cause a potential outbreak of diseases due to the development of a variety of pathogens [75]. Heavy rainfall resulting in overflows carries fecal coliforms to distant areas along with widespread accumulations under soil and sediments [76]. Temperature and humidity also play a key role in the transmission of influenza A virus (H1N1) [77]. It has been identified that transmission of influenza virus occurred more in cold and relatively less humid conditions [78]. Thu et al. analyzed the impact of relative humidity and rainy season over dengue virus propagation in Yangon and Singapore [79]. The results revealed that dengue virus concentration increased remarkably in relative humidity and rainy season in both the regions. 

Some literatures also suggested the involvement of wind in air-borne diseases. Chen et al. performed an experiment using quantitative polymerase chain reaction (qPCR) to find the impact of Asian dust storms (ADS) on influenza virus concentration. The results highlighted that during ADS periods, the concentration of H1N1 influenza virus was substantially higher compared to other days [80].

### 4.2. Impacts on Vectors/Hosts

Ever-increasing human population, unplanned urbanization, restricted habitats, improper land use, and climate are the major factors involved in the emergence and re-emergence of diseases [81]. However, it has also been identified that intrinsic sensitivity of arthropods towards climate change forces the emergence and re-emergence of a variety of infectious diseases [25]. The host can be any living macro-organism such as animals or plants. Vectors act as an intermediate host, which carry and transfer pathogen to host. This subsection explores the possible impacts that climate change and weather can impose on vectors. Past studies have provided enormous evidences of climate change impact over vectors and vector-borne diseases. These vectors are ectothermic in nature and therefore, their vectorial capacity greatly is affected by any change in temperature [82,83,84].

#### 4.2.1. Effect of Spatiotemporal Distribution of Vectors

Temperature rise can lead to a major shift in the habitats of vectors. Vectors living in lower latitude regions can shift their habitat to mid or high latitudes, resulting in geographical amplification of vectors and related diseases. Vector-borne diseases such as lyme, malaria, yellow fever, dengue, and plague are some of the examples with wide geographical expansion in many parts of the world [11]. For instance, snail (*Oncomelania hupensis*) as a vector plays a vital role in the transmission of Schistosomiasis. A study conducted in China found that owing to a continuous rise in winter temperature, *O. hupensis* increased its range with widespread distribution, thereby spreading schistosomiasis over northern China [12]. Another detailed study in Nepal revealed the vulnerability of mountain regions to global climate change. It was found that the rate of warming in the Himalayas (0.06 °C/year) was much greater than the global average warming rate [85]. The consequences of the high rate of warming in these regions were found in terms of spatiotemporal distribution of vector-borne diseases. Firstly, malaria which was confined to the forest and nearby areas of Terai, around 38 districts in Nepal lowlands, has now expanded to mountains covering around 65 districts of Nepal [86,87,88]. In total, 44 species of *Anopheles* mosquitoes were estimated to be affected and among them 7 (i.e., *Anopheles fluviatilis, An. dravidicus, An. willmori, An. minimus, An. pseudowillmori, An. maculatus, and An. annularis*) were responsible for the transmission of malaria. Similar trends were also observed for dengue [89,90,91]. One more vector-borne disease, Japanese encephalitis transmitted by mosquito species *Culex tritaeniorhynchushas,* rose northwards in India and became an endemic in Nepal [13]. Initially reported in 24 districts of the lowland Terai, Japanese encephalitis was transmitted to other mountain regions including Kathmandu [14,15]. Lastly, visceral leishmaniasis and lymphatic filariasis were other vector-borne diseases whose spatiotemporal distribution were reported due to global warming [16,17,18,19]. 

Ticks, another disease-transmitting vector, is found to be greatly affected by global warming in terms of survival and reproduction rates largely due to increase in winter temperatures [92,93]. A recent study in Sweden provided clear evidences for the expansion and distribution of tick *Ixodes ricinus* [94]. From 1990 to 2008, tick’s coverage area expanded almost twice i.e., from 12.5% to 26.8% with major expansion occurring in the northern region. A different study conducted in Russia reported a significant rise in the population of *Ixodes ricinus* over a period of 1977 to 2011 in eastern Tula region [95]. These studies highlighted that the expansions were mainly due to milder winters and extended spring and autumn seasons along with some adverse human activities such as decimation of forest and over exploitation of coal and petroleum reserves, which ultimately promote global warming.

#### 4.2.2. Effect on Mortality Rate

Temperature mostly has a direct influence on the mortality rate of vectors; however, the limited understanding and complexity of the relationship between vectors, disease transmission, and surrounding environment urge more detailed research [96,97]. Among the variety of vectors, mosquitoes have been found to be much more susceptible to threshold temperature limits [98]. For instance, a study conducted in Changsha, China, identified that a temperature of 22–23 °C is optimal for the development of mosquito larvae, while 25–26 °C is suitable for JEV, as it exceeds the breeding temperature, and the tendency of being infected also rises significantly [65]. Recent studies also investigated the impact of different water temperature on the mortality rate of larval and adult dipteran vectors [99,100]. These larval and pupal stages of dipteran vectors are more sensitive to diurnal variations in regional temperature than global variation in temperatures [101]. However, there are two major factors which determine the probability of vector population survival in the geographical regions where climatic conditions surpasses the threshold limits: (a) the capacity and tendency of the vector to espy refuge from extreme conditions; (b) capacity of surrounding environment or vector habitat to provide opportunities for refuges [102].

Among most of the positive effects of extreme climatic conditions on vector’s mortality, there are some vectors whose growth are restricted, thereby a reduction can be observed in the distribution of disease vectors. For instance, viruses responsible for yellow fever and dengue are carried and transmitted by the *Aedes aegypti* mosquito [7]. Previous studies found that the growth of *A. aegypti* larvae ceases when the temperature of habitat water exceeds 34 °C. Moreover, as the air temperature reaches 40 °C, the adult *A. aegypti* concentration also starts to collapse [103]. With a continuous rise in global temperature, disease vectors such as *A. aegypti* may vanish in regions where temperature surpasses their thresholds. Another example includes the case of the *Anopheles* mosquito, vector for *Plasmodium falciparum*, a causative agent of malaria. These mosquito vectors can only survive above 16 °C, thereby on dropping the temperature below it aids control measures over malaria epidemics [104]. 

Precipitation can play a dual role in the distribution of disease vectors, although most of the vector growth accelerates during rainfalls with increasing temperature [105]. A study conducted in Mexico found that rainfall significantly increases rodent population and was responsible for the cocoliztli outbreak in Mexico [106]. However, excessive precipitation can have adverse impacts on larvae growth, as heavy rain may mop off their breeding sites [23]. For example, *Culex,* a mosquito genus which carries the West Nile virus, generally breeds in grubby water pools and drains. Heavy rainfall can wash off the drains, thereby limiting the growth of *Culex* and hence reducing transmission of the West Nile virus [107]. In case of drought, studies have found a contrasting effect on vector’s growth, especially mosquitoes. Adult *Anopheles,* which mostly breeds in clean and stagnant water, were significantly affected by drought due to a severe reduction in breeding sites [108]. Contrary to this, another study on the impact of drought highlighted a reduction in the flow of water in brooks, which ultimately introduces small pools and patches of putrid water that act as perfect breeding sites [109].

### 4.3. Mode of Transmission

Disease transmission can be categorized as direct and indirect transmission. Direct transmission to humans basically includes transmission through physical contact, droplet transfer, and by using infected formites. Indirect transmission to humans mostly occurs with the involvement of vectors or other organisms. Past studies have shown a positive involvement of climate variables and extreme weather conditions in the transmission of diseases, although the transmission mechanism is not well understood yet. This subsection discusses the impacts and consequences of climate change on the transmission of diseases in humans. 

Wind can play an important role in the transmission of air-borne pathogens and viruses. Wind storms and other similar calamities hold great potential for transmission of viruses and pathogens from endemic regions to other distant regions. As an example, it has been proved that Asian Dust Storms (ADS) were involved in a widespread transmission of the H1N1 influenza virus [80]. Some studies even concluded that ADS can carry those influenza viruses from Asia to the Americas during winters through prevailing winds [110]. Wind can also have a dual impact on disease-causing vectors. One example related to all species of mosquitoes is that strong winds can significantly minimize biting opportunities but also provide aid in spatial distribution, thereby increasing disease transmission probability [111].

Climate change can also alter the probability of disease transmission by increasing human-vector and human-pathogens contact. This can be well understood with *Aedes* species bloodmeal digestion patterns, which change with temperature. The quicker this species digests their bloodmeal, the more opportunity they will have to search for and hence increase chances of transmission [112]. For example, at 8 °C, *Aedes* larvae develop in 38 days, whereas at 12 °C, they last only for 18-20 days; bloodmeal digestion generally takes 30 days at 4 °C and just 5 days at 20 °C; in the case of embryonic developments, it takes 42 days at 4 °C whereas only 22 days at 12 °C and just 8 days at 20 °C [112,113]. Sandfly, another vector which can transmit leishmaniasis, an infection caused by *Leishmania* spp. Temperature greatly manipulates the biting activity and maturation of *Leishmania infantum* in infected sandfly [114]. It was found that the biting behavior of sandfly is more common in summer seasons, although too hot and dry conditions can be detrimental for sand flies. Campylobacteriosis, a bacterial gastrointestinal disease is stimulated by thermophilic *Campylobacter* spp. bacteria. Researchers found that broiler and poultry meat were major infection transmission sources [115]. Further study also revealed that the colonization of campylobacter, along with those meats, have an exponential relationship with rising temperature [116]. 

A key element for pathogen transmission and their spatiotemporal occurrence is the Extrinsic Incubation Period (EIP). EIP specifically refers to the time interval between ingestion of pathogen by the fly from an infected person and the release of the same pathogen while feeding another person, thereby causing multiplication and propagation of complex sexual cycles [117]. Temperature greatly influences the tendency and occurrence of EIP [118] i.e., EIP occurs at a faster rate at rising temperatures. Contrary to this, at lower temperatures, EIP duration can be longer than fly life expectancy and therefore, even with abundant vector populations, the pathogen transmission cycles can cease. 

Moreover, during too hot conditions, the mortality rate of fly can overcome the shortening effect of EIP, resulting in the upper threshold limit of temperature for transmission [119]. 

## 5. Need for Urgent Human Response

**Figure 3:**
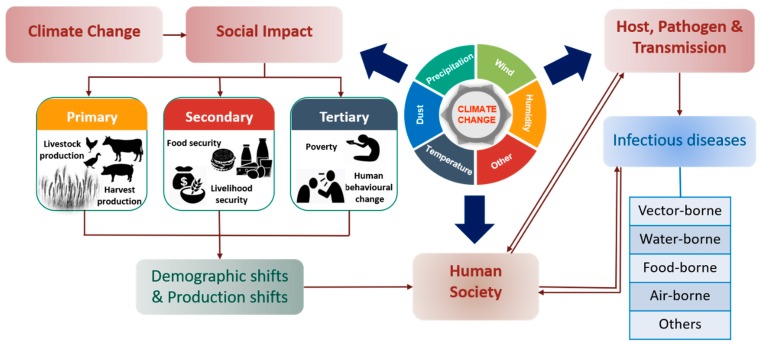
Social impact of climate change affecting livelihood, human society and infectious diseases adapted from [6,7,8,9,10,128,129,130].

Economic and social factors hold a great potential in understanding the changing patterns of climate and associated diseases [10,77,128]. It has been found that certain regions and communities are more vulnerable because of their inability to respond appropriately to weather events posed by climate change [129,130]. The threat of vulnerability can have two aspects: First, measures taken to abate weather and climate-associated risks through awareness programs, figuring out different diseases and biosecurity. Second, availability or access to safe and clean drinking water, hygienic food, and proper sanitation [131,132]. Uncontrolled and unplanned urbanization in India has left many regions highly vulnerable to diseases in wake of climate change. The country has seen a dramatic increase in malaria and dengue cases in the past decades. [133,134]. Likewise, communities with limited access to clean water are more vulnerable to diarrhea, as the ailment occurs more frequently in water-scarce regions [135]. However, an exception can also be seen in well-developed societies who have access to advanced technologies and hold alternative resources to eradicate the challenges imposed by water scarcity. Figure 3 illustrates the outline of social impacts of climate change on livelihood, human society and infectious diseases.

### 5.1. Climate Change and Public Health System

Among all the mitigation measures for reducing the vulnerability of health risks associated with climate change, improved public health system and quality infrastructure are most significant [26]. Underdeveloped and developing countries are more vulnerable to climate change and related health risks. Their inefficient public health systems in terms of resources, infrastructure and capabilities are the major cause of minimal resistance towards elevated health risks. It was the below par performance of public health services and lack of anti-malaria spraying that caused more than 75,000 cases of malaria after a hurricane incidence in 1963 in Haiti [136]. Similar tragedy occurred in Guatemala, Honduras and Nicaragua where dengue and malaria morbidity rates rapidly increased after Hurricane Mitch in 1998 [137]. A similar pattern was observed in the outbreak of *Vibrio* cholera after the earthquake and hurricane due to poor sanitary systems [138]. A striking increase in a group of infectious diseases such as hepatitis, measles, typhoid, malaria and paratyphoid fever were found after five months of hurricane calamity in Dominican Republic in 1979. It was believed that the major causes were crowded dwellings, poor sanitation, unhygienic food and water, and low immunization rates [56]. The outburst of H5N1 in 2006 was also believed to be due to lack of efforts by the Egyptian government towards control and prevention. This ultimately resulted in the shortage and improper supply of commercial H5 poultry vaccines [139]. It is apparent that with quality public health services, advanced infrastructure, public programs and functions, and proper government monitoring, health risks associated with climate change can be potentially minimized. 

### 5.2. Climate Change and Adaptation Measures

Proper adaptation measures against climate change can play a significant role in addressing challenges regarding infectious diseases. Reforestation, proper sanitation, and improved drainage systems are some of the measures recommended by the United Nations Environment Programme (UNEP) in 2013 in African countries in order to mitigate climate change [140]. Hess et al. highlighted two distinct view points on climate change consequences and responses to public health. The first view argues that climate change will enhance the adverse impacts on human health; however, providing increased investment to health-related infrastructure with considerable funding and support can restrict the health impacts. Contrastingly, the second view focusses on the fact that climate change can also affect human health by destabilizing public health systems, which may indirectly pose a threat to infrastructure; thus, a broad understanding is required on adaptation [141,142].

Accurate weather forecast can be considered one of the important adaptation measures through which the public can get ample time to prepare for weather-related health risks. For example, during an El Nino weather event in 1997/98, the Pacific ENSO Application Center (PEAC) warned governments regarding severe droughts and tropical cyclones. Numerous public awareness programs and campaigns were then launched, which resulted in a remarkable reduction in hospital admission cases with diarrhea, malaria, dengue and other vector-borne diseases [110]. Another example is the case in Botswana, where a successful forecast was made regarding the outbreak of malaria [143]. However, these forecasts are not always reliable, considering the confined knowledge of complex weather patterns, particularly extreme weather events [29]. 

### 5.3. Climate Change and Adaptation Policy

Implementation of climate change-related policies or adaptation policies with long-term vision can greatly help in reducing the risks of adverse health impacts [144,145]. Adaptation policy refers to policy design and implementation in order to minimize the risks introduced by climate change [146]. These policies must be designed considering future scenarios and ample flexibility to tackle future complications [145,146,147,148]. A quality adaptation policy must consider governance capabilities over responsiveness, reflexivity, revitalization, and resilience [149]. Most South Asian countries like Nepal, Bangladesh and even India greatly lack effective climate change policies [150].

Recently, researchers also revealed the relative significance of discrete emotions such as hope, interest and worry in designing climate change policies around the world [151]. As a part of future research, it may be interesting to investigate the extent of positive emotions and linked potential solutions to climate change. There might be a considerable possibility that positive emotions matter more than negative emotions cohorted with climate change impacts [151]. A high compassion state will elicit stronger belief among the public that climate-related humanitarian crisis is solely because of anthropogenic activities. This can mediate further support for quality climate change policy designing, especially among political conservatives [152]. 

The main obstacles that influence any adaptation measure revolves around the following six factors that link climatic variables to adverse health impacts: complexity in disease patterns and mortality, food, water and sanitation, extreme events, population, human adaptation measures and capabilities. The following are considered responsible for five major challenges in developing an effective policy response framework: poverty and equity-related, informational, technological, institutional and sociopolitical [144,145,146,147,148,149,150,151,152].

### 5.4. Climate Change and Social Development

Social development in itself can play a vital role in reducing elevated health risks related to climate change. The relative number of infectious diseases after an extreme weather event such as a hurricane or a tropical cyclone are much more in underdeveloped and developing countries when compared to developed nations [153]. In 1998, the Dominican Republic faced widespread acute respiratory infection and gastrointestinal infections due to the occurrence of Hurricane Georges [154]. In addition, Hurricane Mitch of 1998 caused high dengue and malaria morbidity rates due to lack of awareness and substandard health-related infrastructure in Guatemala, Honduras and Nicaragua [137]. In contrast with the earlier-mentioned health issues, a study conducted by Toole revealed that no positive cases with infectious diseases were found in the post-hurricane analysis report [155]. In rural India, around 360 million people do not have access to toilets and more than 600 million people defecate in the open [156,157]. India faced an estimated loss of around $54 billion in 2006 due to health-related issues, out of which 70% were due to issues related to inadequate sanitation systems [158]. Lack of environmental awareness, poor education regarding public health, limited medical resources, financial crises and limitless corruption in underdeveloped and developing countries bind these societies to more health-related challenges. 

## 6. Discussion and Conclusions

Climate change refers to the long-term and sudden fluctuations in weather conditions. These fluctuations can promote a suitable environment for the sustenance of pathogens and vectors, which ultimately result in outbreaks of various human diseases. Climate variables such as temperature, wind, precipitation, and humidity constrain the spatiotemporal distribution of pathogens and their vectors. Any change in these climate variables can result in exponential reproduction, survival and distribution of disease-causing pathogens and intermediate hosts. Hazardous meteorological and weather events in combination have severe impacts on numerous infectious diseases and may increase the complications manifold. Thereby, it becomes imperative to develop relationships between climate change, pathogens, vectors, and related diseases.

Humans are highly vulnerable to the threats of infectious diseases imposed by climate change. Therefore, humans themselves must play a prime role in the adaptation and implementation of proactive measures for the alleviation of adverse health influences of climate change. Researchers have confirmed that the effects of changing climate variables throw more challenges and problems in some communities and societies than others. Hence, instead of global projections, regional projections for climate variables are required for minimizing health implications. In addition, due to lack of resources and abilities, specific areas and certain populations are more prone to the associated risks and challenges. There is the utmost requirement of teamwork between healthcare providers, scientific researchers and governments in developing as well as developed countries to work towards the upliftment of underprivileged societies. This can significantly reduce the vulnerability of incapable societies towards elevated health risks linked with climate change. The exposure of infectious diseases to humans can be reduced significantly through effective adaptation measures and positive human responses against climate change; for example, frequent allocation of required resources related to finance and health care and promotion of awareness programs. Integration of health surveillance with terrestrial and marine monitoring systems may prove more beneficial through advanced satellite imaging and forecasts. Effective early alert systems combined with integrated mapping of consequences, expenses and conditions can facilitate efficient public health mediation with proper designing of policies. Human capability to counter the negative health effects posed by climate change depends heavily on the generation of relevant, reliable and accurate information. Strengthening scientific, technological, and informational capacity within local and regional levels can be crucial for the establishment of a rejuvenated public health movement. This capacity enhancement can significantly reduce vulnerability and induce resilience in infrastructures at local, regional and national levels. 

Researchers pursuing scientific investigations on climate change and related health risks can be primarily divided into three groups: The first group examines complex issues employing experimental and modeling studies to predict the possible relationship between health risks and climate variables—trying to figure out all possible alternatives to minimize the potential risk of human diseases associated with any change in climatic conditions. The second group of researchers seeks to calculate which climatic variables and to what extent may prove beneficial for a group of disease pathogens, vectors and mode of transmission. The last group deals with extreme weather events and related economic losses. This group also thoroughly analyses the elevated psychological health risks post each extreme weather event. The limited communication and lack of collaboration among these sections have resulted in a void between understanding of climatic variables and the forecast of changing the landscape for human diseases. This can also be seen through various contradictions between authors in their outcomes. As discussed earlier, there is the strong need for precise and accurate prediction for any change in climate variables and to timely identify all possible health risks. Therefore, to improve weather predictions and human adaptation capability, all aforementioned research groups should work under the same umbrella. If not, external support should be provided to enhance effective collaboration and effortless communication so that any negative health effect can be minimized. Furthermore, as multidisciplinary and a variety of approaches are involved in the mitigation processes, a much broader view is required to provide in-depth knowledge and understanding of the correlations between climatic variables and human health. For any detailed research, high-quality and precise modeling is a prerequisite. Time series models such as Error Correction Models (ECM), Auto Regressive Moving Average with exogenous variables (ARMAX), and non-parametric forecasting models; panel data and spatial models such as spatial lag and spatial error models, fixed and random effect models, and dynamic panel data models; and non-statistical approaches such as Computable General Equilibrium (CGE), Comparative Risk Assessments models (CRA), and Integrated Assessment Models (IAMs) are some of the models that need to be properly used wherever required. This will not only help in converging the gap in the present state of understanding but can also provide more realistic data suitable for accurate predictions for any consequences. 

The following three levels of practices need to be adapted for successful vindication of adverse impacts of climate change on disease occurrence: (a) improved and stringent policies must be implemented to reduce carbon emission by utilizing renewable resources and to promote carbon utilization by employing carbon dioxide sequestration technologies such as bio-sequestration or chemical sequestrations technologies, (b) detailed research is needed to identify all possible acquaintances between climatic variables and the emergence and re-emergence of infectious diseases, (c) appropriate infrastructure with improved public health systems should be available to successfully encounter adverse outcomes at the regional, state and national levels. We hope that the present review work can provide a cause for more multi-disciplinary projects and collaborations, which can converge the gap in understanding the complicated relationship between climate change and induced health risk.

Climate change is reported to be responsible for millions of deaths and billions of major or minor disabilities since its start in the 1970s [159,160]. The Intergovernmental Panel on Climate Change (IPCC) also predicted that the existing human health risks will be exacerbated in the coming decades [161]. Climate change-associated dilemmas such as droughts, floods, heatwaves, fires, water and vector-borne diseases, water scarcity, and shortage of food pose serious threats and will continue to target more vulnerable groups like the poor, children, pregnant women and the elderly [162,163]. In this century, adverse health impacts due to climate change will continue to rise; a few diseases may disappear, but a majority will emerge and transmit more rapidly. The most effective approach to reducing climate change induced health risk is through adaptation of proper measures, which can be identified through scientific investigations and social advances. Scientific understandings are required to move beyond the empirical observations to connect the links between climate shift and emerging diseases, and to reach more reliable conclusions. These approaches need accurate information about the climate change related outcome of health implications. Moreover, advanced modeling and simulation techniques are required for proper long-term analysis of climate change-based spatiotemporal processes. Being dexterous in figuring this arduous process can be the foundation of accurate prediction of health consequences of climate change and the espousal of efficient adaptation measures. Finally, for such advancements, new protocols will be required for safe and effective sharing of information, resources, and awareness campaigns.

## Figures and Tables

**Table 1:** Key studies highlighting the impacts of extreme weather events on human diseases.

Extreme Weather Events	Main Outcomes	References
El Nino	Found responsible for the outbreak of Malaria in Peru, Bolivia and Ecuador due to extreme rainfall in 1983. The effect of El Nino varied by region. San Francisco and Los Angeles evidenced 30–50% rise in hospitalization with a decrease of 5 °C in minimum average temperature due to influenza epidemics. However, 25–40% hospitalizations were observed in Sacramento with just 5 °C in maximum average temperature.	[30] [56]
La Nina	La Nina events cause outbreaks of Japanese encephalitis and West Nile Virus. Occurrence of drought due to the La Nina event was linked to the chikungunya fever epidemic	[30] [57]
Quasi-Biennial Oscillation (OBQ)	OBQ was confirmed as the main cause for the emergence of Ross River virus in Queensland.	[58]
Drought	Among various categories of drought, agricultural drought is the most severe, and many farmers suffer from mental disorders due to significant losses in crop production. Many end up committing suicide.	[59]
Heat Waves	A positive relationship was observed between hospital admission, excessive heat and humidity due to the emergence of angina pectoris. On an annual basis, more than 400 children in the United States die of heatwave-related sicknesses such as diarrhea, typhoid and jaundice. Recent studies provide reliable evidence on the direct relationship between heatwaves and myocardial infarction with congestive heart failure.	[42] [47] [48,49]
Flood	A direct association was observed between gastroenteritis and intensity of flood at Lewes in Southern England.	[60]
Hurricane	Honduras and Venezuela evidenced malaria and dengue outbreak post a hurricane event.	[6]
Cyclone	Cyclone events remarkably increase leptospirosis and cholera incidences.	[52,53]

**Table 2:** Collection of key studies representing the impacts of climate change on pathogens, vectors and transmission of infectious diseases.

Climate Impacted Variables	Emerged Diseases	Main Outcomes	References
***Pathogens***			
Japanese Encephalitis Virus (JEV)	Japanese encephalitis viral disease	A temperature range of 25–26 °C is best for transmission of JEV through mosquitoes.	[64,66]
Chikungunya Virus (ChikV)	Chikungunya viral fever	Mild winters, summers times with temperature around 20 °C and average rainfall (>50 mm) is optimum for ChickV transmission.	[120]
*Campylobacter* spp.	Campylobacteriosis	Lower surface water temperatures are more favorable for their growth. At higher water temperatures and more intense UV radiations, other bacteria can out-compete them, which can lead to extinction of *Campylobacter* spp.	[121]
Influenza Virus H5N1	Bird flu/Avian Influenza	H1N1 influenza virus concentration was found to be significantly higher during Asian dust storms compared to other days.	[80]
Influenza A virus H1N1	Influenza (flu)	Temperature and humidity enhance the transmission of influenza A virus (H1N1).	[77]
Dengue virus	Dengue	The impact of relative humidity and rainy season over dengue virus propagation were found to be responsible for the outbreak of dengue in Yangon and Singapore.	[79]
*Leishmania* spp	Leishmaniasis	Temperature greatly influences diapause and maturation of *L. infantum* in infected sandfly.	[114]
***Vectors***			
*Plasmodium falciparum* (Mosquito)	Malaria	P. falciparum grows at a faster rate in warmer temperatures i.e., it takes 26 days for incubation at 20 °C but only 13 days at 25 °C.	[7]
*Anopheles fluviatilis;* *An. dravidicus;* *An. willmori;* *An. minimus;* *An. pseudowillmori; An. maculatus;* and *An. annularis*	Malaria	As a consequence of the rising rate of warming, significant spatiotemporal distribution of vectors was observed in Nepal. Malaria, which was earlier confined to forests near Tarai lowlands (in 38 districts), has expanded to further 68 districts.	[86,87,88]
*Culex tritaeniorhynchushas*	Japanese encephalitis	Rising rate in warming forces Culex to find refuge and hence has expanded from the northern part of India and became endemic in Nepal.	[13]
*Aedes aegypti*	Dengue	The continuous rise in temperature and changes in precipitation pattern accelerates the growth of *A. aegypti.*	[122]
		Precipitation can modulate the size, behavior and population of *A. aegypti*. With rainfall (>50 mm), significant cases of dengue were seen, whereas a temporary decrease in cases was seen during extreme rainfall.	[123]
		A significant spatiotemporal distribution of mosquitoes was observed over a wide area due to rise in warming rate.	[89,90,91]
		The growth of *A. aegypti* ceases when water temperature surpasses 34 °C and for the adult one if the temperature exceeds 40 °C.	[103]
*Aedes* spp	Malaria, Dengue, Chikungunya	Effect of temperature on *Aedes* species activities: *Larvae development:* 38 days at 8 °C and 18 days at 12 °C. *Bloodmeal digestion:* 30 days at 4 °C and 5 days at 20 °C. *Embryonic developments:* 42 days at 4 °C, 22 days at 12 °C and 8 days at 20 °C.	[112,113]
*Aedes* spp.; *Haemagogus* spp.; *Sabethes* spp. (Mosquito)	Yellow Fever	Warmer climate encourages the growth of mosquito species, resulting in outbreaks of yellow fever in Africa and South America.	[124]
*Culex* spp. (Mosquito)	West Nile Fever	The aggressiveness of Culex spp. was found to be strongly correlated with humidity, rainfall and temperature changes.	[125]
*Ixodes Ricinus* (Tick)	Tick-borne encephalitis	Faster development and increased activity with rise in humidity and temperature.	[20]
		Due to mild winters and extended spring and autumn, *I. ricinus* coverage area increases from 12.5% to around 26.8% in Sweden during 1990–2008.	[94]
*Oncomelania hupensis* (Snail)	Schistosomiasis	With continuous rise in winter temperature, *O. hupensis* widens its distribution range, thereby spreading schistosomiasis in northern China.	[12]
***Transmission***			
Influenza Virus H5N1	Bird flu/Avian Influenza	The H5N1 outbreak is associated with wild fowl migration.	[126]
		H5N1 viruses were found to be carried away to distant areas during Asian dust storms.	[80]
Hantavirus	Hantavirus pulmonary Syndrome	During extreme conditions like floods, deer mice may approach human dwellings in search of food and can transmit disease.	[127]
*Leishmania infantum*	Leishmaniasis	The biting activity of sand fly is found to be more common in summer months, although too hot and dry conditions are not suitable for survival of sand flies.	[114]
*Campylobacter* spp.	Campylobacteriosis	Colonization of campylobacter along with those meats have an exponential relationship with rising temperature.	[115,116]
